# Gastrentrological clinical trials in Italy: an association study on regional economic factors and healthcare system efficiency

**DOI:** 10.3389/fphar.2025.1594504

**Published:** 2025-08-26

**Authors:** Maurizio Polignano, Davide Guido, Giuseppe Dalfino, Gianluigi Giannelli

**Affiliations:** National Institute of Gastroenterology, IRCCS “Saverio de Bellis” Research Hospital, Castellana Grotte, BA, Italy

**Keywords:** clinical trials, gastrointestinal oncology, Italian NHS, regional disparities, economic factors, healthcare efficiency, IRCCS

## Abstract

**Introduction:**

This study investigates the geographical distribution of gastrointestinal oncology clinical trials (CTs) in Italy and their association with regional economic factors and healthcare system efficiency. Despite the Italian National Health Service (NHS) providing universal healthcare, significant disparities exist, particularly between wealthier northern/central regions and less affluent southern regions. The study examines how socio-economic parameters, NHS funding, and specialized research institutions (Institutes of Hospitalisation and Care of a Scientific Character, IRCCS) influence CT accessibility and distribution.

**Methods:**

A retrospective analysis of 103 active interventional CTs (March 2020–March 2024) registered on ClinicalTrials.gov was conducted. Socio-economic and healthcare data, including population density, NHS expenditure, regional income, and unemployment rates, were sourced from national databases. Spearman’s association and Poisson regression analyses evaluated associations between CT numbers and regional variables for Italy’s 20 regions.

**Results:**

CT distribution was highly clustered, with 44 % conducted at IRCCS facilities, predominantly in northern/central regions. Strong associations were observed between CT numbers and NHS expenditure (ρ = 0.913, p ≤ 0.001) and population density (ρ = 0.777, p < 0.001). Southern regions and islands hosted fewer trials, reflecting lower healthcare funding and IRCCS availability. The total number of IRCCS facilities (both public and private) showed a strong positive correlation with the number of CTs (ρ = 0.837, p < 0.001). Regression models identified population size, density, and health expenditure as significant predictors of CT numbers, while unemployment showed an inverse relationship. No association was found with public pharmaceutical expenditure.

**Discussion:**

Regional disparities in CT accessibility are driven by economic inequality and uneven healthcare infrastructure. Targeted policies to increase funding, expand IRCCS networks in southern Italy, and promote equitable resource allocation are urgently needed. CT distribution may serve as a novel indicator of healthcare system performance. Addressing these imbalances is vital to ensuring equitable patient access to innovative therapies and optimizing the NHS’s research capacity nationwide.

## Introduction

On 23 December 1978, Law No 833, established the Italian National Health Service (NHS), based on universal healthcare, solidarity financing through general taxation and equitable and universal access to healthcare. The NHS, which has been highlighted in international reports, has achieved satisfactory levels of efficiency and excellent standards of care in many regions over the last 40 years (OECD, 2021a; WHO, 2016). However, the NHS is currently facing some major challenges: (i) Italy is experiencing progressive ageing of its population with increasing health needs; (ii) the recent and continuous cuts in public spending are reducing the budget for social care; (iii) the NHS is facing the challenge of increasing costs of healthcare. It is of great importance to ensure that ongoing efforts to contain health system costs do not affect healthcare quality. In addition, monitoring the essential levels of care (Livelli Essenziali di Assistenza, LEA) highlights significant differences in healthcare delivery among Italian regions (Signorelli et al., 2020; Signorelli et al., 2017) that, in turn, contribute to the burdensome migration of patients to best-performing regions (Nante et al., 2021). In the new model of governance of the Italian healthcare system, decisions concerning the funding of the system are made within the State-Regions Conference through the so-called State-Regions Agreements (Intese Stato-Regioni) (Gazzetta Ufficiale, 2003) (art. 8, co. 6, Law n. 131/2003).

In this framework, a Legislative Decree (no. 56/2000) (Gazzetta Ufficiale, 2000) launched a complex reform process aiming at introducing in the healthcare system principles of fiscal federalism, leading to a system of financing of the Regions based on their fiscal capacity and adjusted by equalizing measures. This is a complex process that has long remained unimplemented and has been relaunched in recent years (Legislative Decree 68/2011) (Gazzetta Uffici ale, 2011). To date, healthcare is financed from different sources: i) General taxation of the Regions; ii) Co-participation of the special statute Regions and the Autonomous Provinces of Trento and Bolzano; iii) Internal revenues of National Health Service public healthcare institutions, e.g., tickets.

The State budget essentially obtains funds through VAT and excise duties on fuel and through the National Health Fund (of which the main share is allocated to the Sicilian Region).

Another peculiarity of the Italian NHS is the pluralism of healthcare providers, that are both public and private (Toth, 2016). The latter must receive institutional accreditation by the regional health governance to deliver health services which are ultimately reimbursed within the NHS. The institutional accreditation certifies that providers meet the structural, technological and organizational targets required under current regulations. The 1992 reform also established a “quasi-market” system, based on patients’ choice, a managerial organisation of territorial assistance and general hospitals, and the challenging competition between public and private providers to improve the quality, safety and efficacy of healthcare services (Simonet, 2011).

Despite some concerns, the Italian NHS currently provides relatively high standards of healthcare, as demonstrated by the fact that The Healthcare Access and Quality (HAQ) Index, which measures the quality and accessibility of healthcare services, placed Italy in the top 10 globally in 2016 with a score of 95 out of 100 (GBD, 2016 Healthcare Access and Quality Collaborators, 2018). This high ranking reflects Italy’s strong performance in providing accessible and high-quality healthcare services to the population.

According to the 2023 CEOWORLD magazine Healthcare Index, Italy ranked 30th overall (CEOWORLD Magazine, 2023). This index evaluates healthcare systems based on factors such as quality, science and technology, and economic sustainability. While Italy’s ranking in this index is lower compared to some other assessments, it highlights areas where improvements can be made, particularly in innovation and fiscal management. Moreover, Italy ranked second behind France on the WHO measure of overall health system performance among 191 countries across the globe (Reinhardt and Cheng, 2000). Furthermore, life expectancy of Italian citizens is among the highest in the world, second in Europe after Spain, despite disparities across regions and socioeconomic groups. According to the most recent data, in 2023 life expectancy in Italy was 82.2 years for the general population (+2.8 years from 2020), 80 years for males and 84.3 years for females (WHO, 2024). Italy’s healthcare system is decentralized, as the regions have significant autonomy over their healthcare budgets and policies. This decentralization leads to disparities in healthcare quality and access between different regions. Wealthier regions in the north tend to have better healthcare services compared to the less affluent southern regions. These disparities are influenced by regional economic conditions, local governance, and the efficiency of resource allocation (Sanmarchi et al., 2022).

Clinical trials (CTs) provide critical data, which are fundamental for several types of evaluations such as the efficacy and effectiveness of drugs and diagnostic tools (such as IVD), medical devices, and the methods of treatment, prevention and particularly medicines for treating diseases or just alleviating symptoms of diseases. (LaFleur et al., 2004; Shen et al., 2011). There are important healthcare benefits connected to CTs for national and local economies in developed and developing countries (Park and Vonortas, 2022). These benefits include the recruitment of staff, contractors, and free treatment for thousands of patients who participate in CTs. An analysis of costs for healthcare facilities avoided thanks to the conduction of clinical studies in an Italian Institute of Hospitalisation and Care of a Scientific Character (IRCCS) show that, in relation to a sample of five drug companies participating in a 2018–2020 analysis, out of a total of 235,102.46 €, identified as direct investment, 628,158.21 € of avoided costs were estimated for the NHS, with an additional saving (leverage effect) for the NHS of 3.67 € for each € invested by the companies promoting clinical trials (Polignano et al., 2022).

The development of a novel pharmaceutical product, from molecule to marketing approval, is a lengthy, costly, and high-risk process, often taking 10–15 years or more. With approval rates for medicines entering clinical development below 12% and 75% of the costs tied to clinical trials (phases I–IV) (CEOWORLD Magazine, 2023), the financial burden is immense. Post-approval research and development further escalate costs, with estimates suggesting a full product lifecycle cost of nearly $3 billion per approved drug. Pharmaceutical companies play a central role in funding and supporting clinical trials (Berdigaliyev and Aljofan, 2020), with many scientists involved in these activities either employed by or receiving grants from these companies. This reliance on industry funding raises questions about transparency and potential biases in trial design and reporting, highlighting the need for robust oversight and alternative funding mechanisms.

In this context, indirect indicators can serve as valuable tools to assess the broader economic and social impacts of pharmaceutical innovation. When direct data on the societal benefits of new drugs—such as improved public health outcomes or reduced healthcare costs—is difficult to obtain, indirect indicators like employment rates in the pharmaceutical sector, regional economic growth linked to research hubs, or patient access to medications can provide insights. Similarly, these indicators can help policymakers evaluate the effectiveness of regulatory frameworks, identify disparities in drug availability, and measure the economic ripple effects of pharmaceutical investments. By leveraging indirect indicators, stakeholders can better understand the complex interplay between drug development, economic health, and societal wellbeing, ultimately supporting evidence-based decision-making in healthcare and beyond (GBD, 2019 Diseases and Injuries Collaborators, 2020; Nolte and McKee, 2004; Martin et al., 2020).

Indirect indicators are used to measure economic or social phenomena by evaluating related factors when direct data is unavailable or difficult to obtain. These indicators provide valuable insights into complex systems, helping policymakers identify areas for improvement (Nolte and McKee, 2004; Martin et al., 2020). In economics, for example, unemployment rates and *per capita* income are often used as proxies for economic health and regional disparities (OECD, 2021) (OECD, 2021b). Indirect indicators are essential for understanding complex issues and making evidence-based decisions.

So, indirect indicators of the functioning of the regional healthcare system may include.

i. Avoidable mortality rate: mortality from causes preventable with timely and effective care (Nolte and McKee, 2004) (Nolte and McKee, 2004).
ii. Hospitalization rate for ambulatory care-sensitive conditions: admissions for conditions manageable in outpatient settings (Rocha et al., 2021)
iii. Waiting times for consultations and tests: indicator of efficiency and accessibility (Martin et al., 2020).
iv. Patient satisfaction: Measured through surveys (Pedersen et al., 2023).
v. Participation rate in screening programs: Indicator of prevention and access to services (Martino et al., 2024).
vi. Per capita healthcare expenditure: Reflects investment in care (OECD, 2021) (OECD, 2021b).
vii. Regional disparities in access to cares (Bruzzi et al., 2022).
viii. Measure of migration rate (Campostrini et al., 2019).
ix. Quality of healthcare measured by staff training (Worsley et al., 2016).

These indicators offer an indirect perspective on the efficiency and quality of the regional healthcare system and are extensively utilized in economic evaluations.

Gastrointestinal (GI) cancers represent a major group of pathologies fully spread on Italian territory, with a rich activity in clinical research. For instance, The Italian GBD Initiative, a network of researchers contributing to the Global Burden of Disease Study, assessing mortality, incidence, and prevalence of diseases, including gastrointestinal (GI cancers. GI cancers are a major group of pathologies widespread in Italy, with significant public health impact and active clinical research involvement (GBD 2019 Study) (DATABASE ISTAT, 2023).

The objective of this study is to evaluate the number of interventional pharmacological clinical trials as a novel indicator of the efficacy of the Regional Health Services (RHS). In fact, it is representative of both regional socio-economic characteristics (e.g., average *per capita* wealth) and the functioning of the RHS (e.g., annual costs and government funding).

## Materials and methods

### Trial data

A retrospective review of active interventional clinical trials (both profit-making and non-profit) in Italy in the field of gastrointestinal neoplasms was conducted between 01/03/2020 and 01/03/2024. The search was conducted using the 'clinicaltrials.gov' database, by searching using the keyword “neoplasm”, and filtering by phase of study (from one to 4), study type: interventistic; then reviewing all CT, selecting only GI clinical trials. Out of the 962 trials initially resulting from the search (see Supplementary Material, Table 1), the search finally yielded 103 trials (Supplementary Material, Table 2). The selection of interventional trials was made *a priori*, as they offer treatments and therapies that are not part of standard clinical practice (Regulation (EU)), due to their definition. These trials cover the main gastrointestinal oncological diseases. For each study, the centres in Italy at which they are active were extracted. Studies are multicentric and active in a total of 630 Clinical Centers. For each centre, city, region and postal code (postcode) were identified. Notably, each Center can have several different studies active at the same time.

**TABLE 1:** Overview of key socio-economic and healthcare-related parameters used to analyse regional disparities in Italy. CU: official document that summarizes an individual’s income and tax-related information for a specific fiscal year; ISTAT: Italian Statistics Agency, MEF: Ministry of Economic and Finance.

Parameter	Description
Regional population	The regional resident population is calculated by gender, year of birth and marital status on 31 December of each year by Italian statistic Agency (ISTAT)
Population density	Population density is expressed as the ratio between the number of people living in each area and the area of that area. The data are taken from the Italian Statistical Yearbook, published by ISTAT.
Average annual income	The establishment of the statistical database is predicated on the consideration of income, IRAP and VAT declaration forms for all categories of taxpayers. In addition, for natural persons only, the processing of 730 and CU (Certificazione Unica) forms is undertaken as published by ISTAT.
RHS expenditure	Current healthcare expenditure (CHE) per region refers to the total spending on healthcare goods and services within a specific region during a given period, including both public and private funds. It covers expenses such as hospital care, outpatient services, medications, and preventive care, but excludes capital investments. Data are published by Italian MEF.
RHS’ public financing	Effective healthcare expenditure financing per region refers to the actual financial resources allocated to cover healthcare costs within a specific region, including both public funds (e.g., state and regional budgets) and private contributions (e.g., out-of-pocket payments or private insurance). This indicator represent the capacity of the Regional Health Service (RHS) to deliver health services in an efficient and appropriate manner, while respecting the budget constraints derived from ordinary state funding and from the General Social Security Entities' (GSEs) own revenues. Data are published by Italian MEF.
Pharmaceutical expenditure	Refers to the total financial resources allocated for the purchase of medications, including prescription drugs, medical devices, in-vitro-diagnostic and other pharmaceutical products. It encompasses public spending both hospital and territorial services, published by Italian MEF.
Unemployment rate	ratio of job seekers to the corresponding labour force labour force published by ISTAT.

**TABLE 2:** Distribution of clinical trials across Italian regions (period 2020–2024).

*Region*	*N. of CT*
Liguria	14
Lombardy	179
Piedmont	29
Valle D’aosta	0
Emily Romania	63
Friuli Venezia Giulia	9
Trentino A.A.	0
Veneto	73
Abruzzo	1
Lazio	46
Marches	10
Tuscany	72
Umbria	1
Basilicata	4
Calabria	3
Campania	59
Molise	0
Apulia	39
Sardinia	11
Sicily	17

### Socio-economic data

The parameters employed in the analysis are delineated in Table 1 and will be discussed below.

### Comparative analysis of the wealth of the Italian regions

To assess the distribution of clinical trials across the territory with respect to regional socio-economic capacities, we performed an analysis of income and wealth distribution across Italy to compare the average wealth “pro capite”. Data for the year 2022, the latest currently available, are from the Eu-Silc (European Union Statistics on Income and Living Conditions) project, carried out in accordance with Regulation of the Parliament Regulation No. 1177/2003 and from the 2021 application of EU Regulation 2019/1700), one of the main data sources for the European Union periodic reports on the social situation and the extent of economic hardship in the member states. (Regulation EU, 2019). The indicators provided by the Regulation focus on income and social exclusion, in a multidimensional approach to the problem and with a particular focus on aspects of material deprivation.

### Comparative analysis of the average unemployment rate in the Italian regions

To assess the distribution of clinical trials across the territory with respect to regional socio-economic capacities, the official unemployment data provided by the National Statistics Authority (ISTAT) for the year 2022 were evaluated. Unemployment rate is a measure of the prevalence of unemployment in an economy system. It is calculated as the percentage of the total labour force that is unemployed but actively seeking employment and willing to work. The survey is regulated by specific acts of the Council of the European Commission, the main one being (Regulation, 2014) 2019/1700 of the European Parliament and of the Council (Regulation EU, 2019).

### Comparative analysis of economic parameters of the Italian NHS

Evaluating the performance of the Italian National Health Service involves analysing various economic parameters to ensure efficiency, effectiveness, and sustainability. We have evaluated, for the year 2022, Health expenditure, pharmaceutical expenditure and ratio of pharmaceutical expenditure to health expenditure for the Italian health services for each Italian region. The data were extracted from the annual report of the Italian Ministry of Economy and Finance (MEF) (Rapporto, 2023). Data were correlated with the distribution of clinical trials by Italian Regions.

The source data for all these analyses are provided in the Supplementary Material and are, also, fully accessible to the public.

### Economic parameters of the Italian NHS

Evaluating the performance of the Italian National Health Service involves analysing various economic parameters to ensure efficiency, effectiveness, and sustainability. We have evaluated, for the year 2022, Health expenditure, State financing of the health service, pharmaceutical expenditure and ratio of pharmaceutical expenditure to health expenditure for the Italian health services for each Italian region. The data were taken from the annual report of the Italian Ministry of Economy and Finance (MEF) (Rapporto, 2023). Data were correlated with the distribution of clinical trials by geographical area (both regional and macro-areas). The source data for all these analyses are provided in the Supplementary Material and are fully accessible to the public.

### Role of the Italian IRCCS

Italian IRCCSs (Institutes of Hospitalisation and Care of a Scientific Character) are of pivotal significance in the domains of biomedical research and advanced care delivery, effecting a synthesis between healthcare and scientific innovation. Key functions encompass the conducting of clinical trials, the development of innovative therapies and the training of specialised personnel. They function as national and international reference centres, thereby promoting scientific collaborations and technology transfer. An investigation was conducted into the role of the parties in the distribution of CTs across the country, with particular reference to association parameters.

### Statistical analysis

The present study examines the regions of Italy (n = 20, statistical units), each of which has unique characteristics in terms of economy, population, infrastructure, and environment. The employment of public available datasets, facilitates the analysis of data, including population density, economic activity, cost, and costing of Regional Health Services. This methodological approach has been instrumental in identifying patterns, disparities, and trends, including the north-south economic divide and the variation in coastal versus inland regions. The analysis of the 20 Italian regions was conducted, encompassing the following assessments for each region: i) average wealth “pro capite” (based on ISTAT 2022 data) (DATABASE ISTAT), ii) expenses for RHS (based on MES 2022 data (Rapporto, 2023)), and iii) population and density (based on ISTAT 2022 data (DATABASE ISTAT)).

Particularly, the statistical analysis conducted in this study was primarily based on the Spearman’s association (ρ) to explore the relationships between the number of active clinical trials and various socio-economic and NHS healthcare parameters and IRCCSs presence on regional basis. The study focused particularly on regional disparities in Italy. The study examines associations between the number of CTs activated during the observation period and the registered socio-economic factors for 2022, which is the most recent data available at the time of writing. To facilitate the analysis, it was hypothesised that these parameters remained relatively stable between 2020 and 2022. Descriptive statistics were evaluated as median and interquartile range were determined for all quantitative variables.

In addition, Poisson regression models were also applied to evaluate the regional mean effects of regressors as i) population (i.e., number of regional inhabitants in 2022), ii) population density in 2022, iii) regional unemployment (%) in 2022 and iv) EC current health expenditure in 2022, on the number of clinical trials performed. Of note, the dichotomous IRCCS indicator was not consider as regressor because in all the region without IRCCS no clinical trial was active (i.e., lack of variability).

The regression analysis is based on geographical distribution of clinical trials on regional areas. The analysis employed Poisson regression models (involving bias reduction to address the relatively small sample size) to assess associations between regional predictors and a count-based outcome, using data from 20 observational units (likely regions or districts). Predictors included inhabitants (scaled per 100,000 population), population density (inhabitants/km^2^), unemployment (%), and health expenditure (per €1000). Incidence rate ratios (IRRs) were calculated to quantify the change in outcome rate per unit increase in each predictor, with 95% confidence intervals (CIs) and p-values evaluating statistical significance.

To improve the effect interpretation, the exponential transformation of the slope coefficient associated to regressor allowed more easily interpretable rate to be obtained on which P-values and 95% CIs (confidence intervals) were calculated.

The calculations were conducted in Microsoft Excel (Version 16.93.2) for MacOS and R software 4.4.2 (R Core Team, 2024) on Windows 11 OS (build 22,631.4602). Finally, R/brglm2 package (R Core Team, 2024; Kosmidis, 2023; Kosmidis and Firth, 2021; Kosmidis et al., 2020) was also used to address small sample size issues.

## Results

The study stemmed from the observation of a strong connection between the geographical distribution of clinical trials and macroeconomic parameters used to describe the functioning of the NHS in Italy.

**FIGURE 1:**
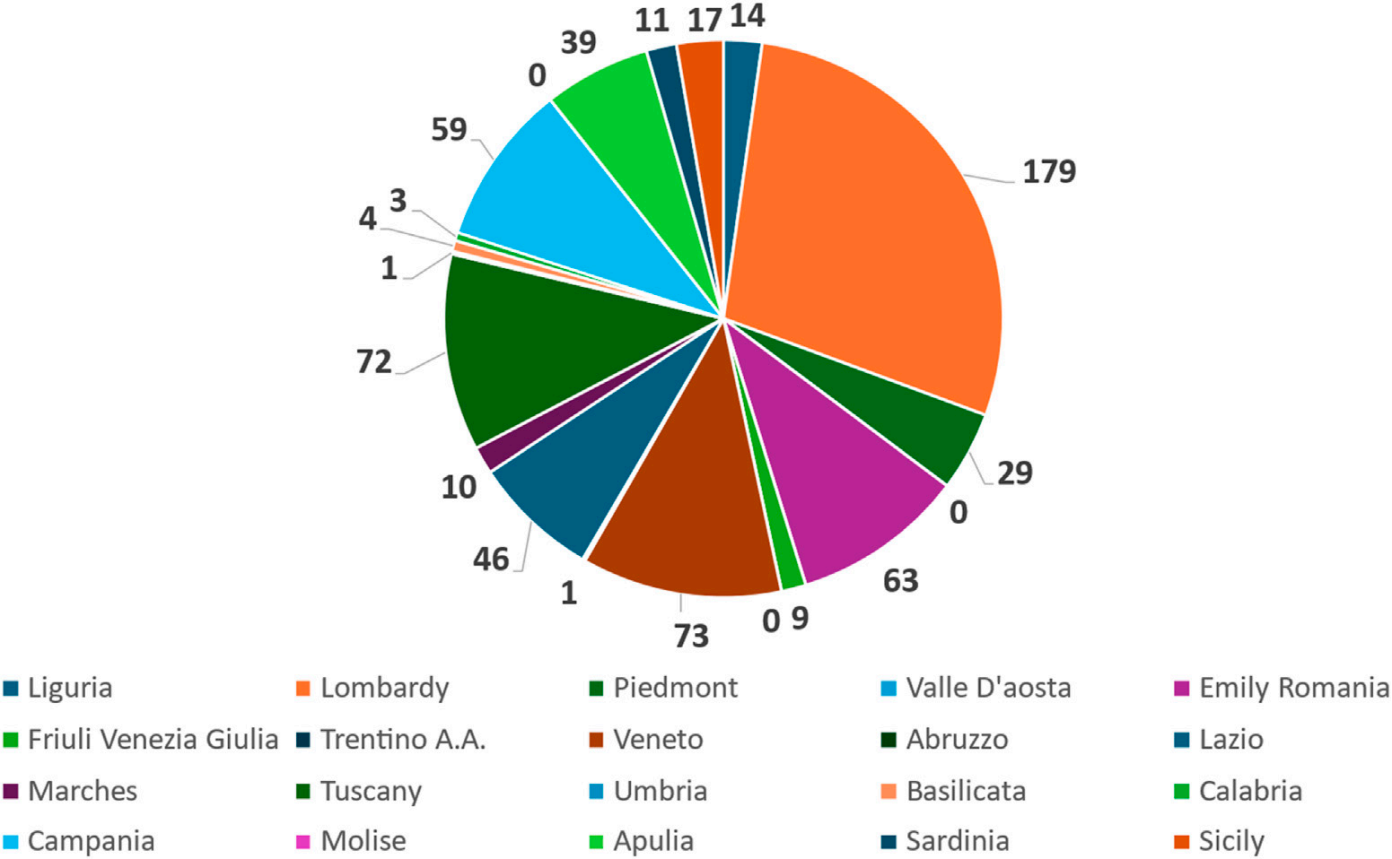
Pie chart representing the distribution of active interventional gastrointestinal oncology clinical trials across the 20 Italian regions. Each slice corresponds to the absolute number of clinical trials identified per region, as retrieved from ClinicalTrials.gov.

These CT are distributed over each region as indicated in Table 2. Data are graphically plotted in Figure 1.

The distribution of clinical trials (n = 630) across Italian regions is not balanced. It is irregular, demonstrating a few regions with significantly higher numbers of trials compared to the majority. We have also calculated Median value = 12.5 and an Interquartile Range (IQR) = 50.5 for this distribution. To verify the association, we correlated the number of clinical trials divided by regions and two different classes of descriptors: one related to the peculiar characteristics of the territory and another to the level of NHS funding. Also, the number of IRCCSs was evaluated.

As far as the descriptors of the economic capacity of the territory are concerned, the parameter of annual wealth produced (2022 data), assessed by individual region, was used (Supplementary Material, Table 3). For each Italian region, we tabulated the average annual income, the number of inhabitants, the area of the region and the number of active CTs. From these data, we calculated the ratio between the number of CTs and the resident population, expressed as number of trials/100k inhabitants. Also, the population density, expressed as number of inhabitants per km^2^ of regional area was calculated. Data are shown in Table 3.

**TABLE 3:** The table provides an overview of the regions in Italy, grouped by area (North-West, North-East, Center, South, and Islands). N.b.: Valle d'Aosta, Trentino-Alto Adige, and Molise are not included in the dataset, because does not have any CT activated. CT: Clinical trial.

Region	N. Of CT	Trial/100k Inhabitants	Income (in €)	INHABITANTS	Density of population
LIGURIA	14	0,93	34,487	1.508.847	279
LOMBARDY	179	1,79	41,428	10.020.528	420
PIEDMONT	29	0,68	36,131	4.252.581	167
EMILY ROMANIA	63	1,41	42,278	4.455.188	198
FRIULI VENEZIA GIULIA	9	0,75	37,527	1.195.792	151
VENETO	73	1,50	40,548	4.851.972	264
ABRUZZO	1	0,08	30,634	1.269.963	117
LAZIO	46	0,80	34,957	5.720.272	332
MARCHES	10	0,67	38,160	1.484.427	159
TUSCANY	72	1,96	39,537	3.664.798	159
UMBRIA	1	0,12	41,652	854.378	101
BASILICATA	4	0,75	30,420	533.636	53
CALABRIA	3	0,16	26,603	1.838.150	121
CAMPANIA	59	1,06	28,758	5.590.076	409
APULIA	39	1,00	31,214	3.890.250	199
SARDINIA	11	0,70	28,591	1.569.832	65
SICILY	17	0,35	28,483	4.794.512	186

**FIGURE 2:**
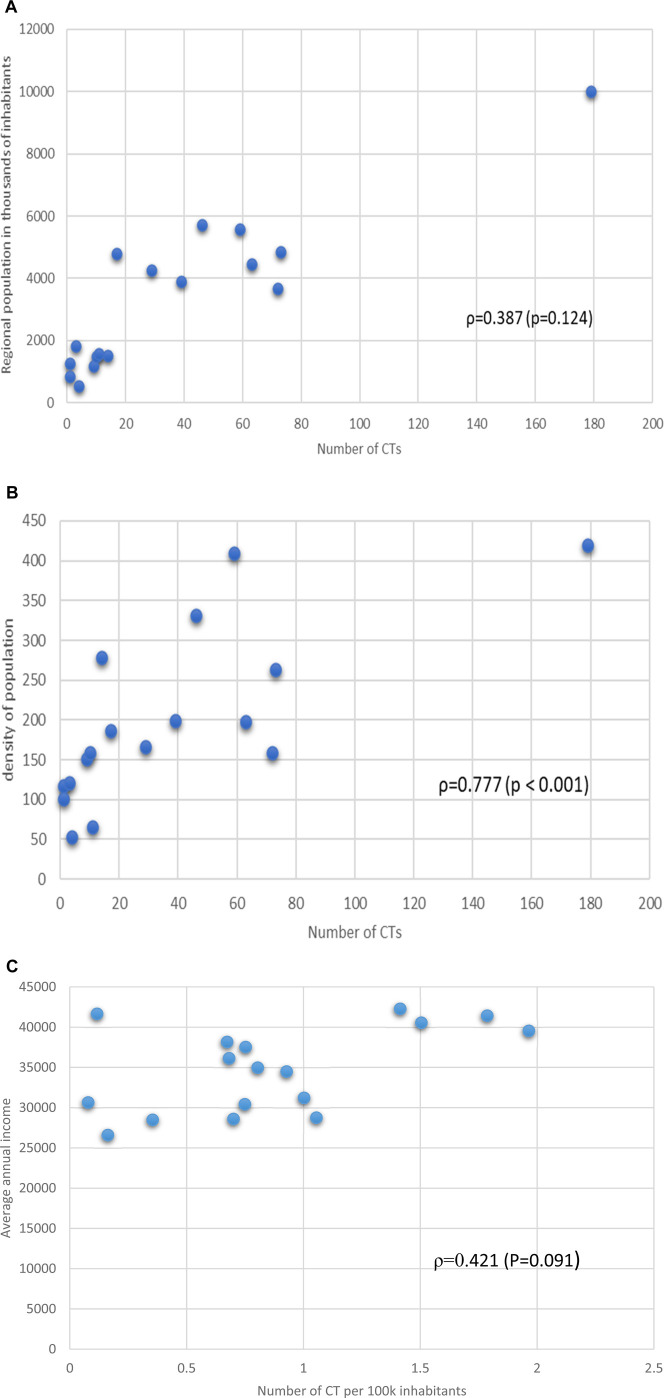
**(a)** Relationship between regional population in thousands of inhabitants and number of the number of CTs. Spearman’s association value and p value are shown. **(b)** Relationship between density of regional population and number of CTs per Region. Spearman’s association value and p value are shown. **(c)** Relationship between Regional average annual income and number of CTs per Region. Spearman’s association value and p value are shown.

We, then, determined the relationship between the number of active trials and the values of territorial parameters, obtaining the following results (expressed as Spearman’s association ρ): Regional population vs. number of CTs ρ = 0.387 (p = 0.124) (Figure 2a), regional density of population vs. number of CTs ρ = 0.777 (p < 0.001) (Figure 2b), Average annual income vs. number of CTs per 100k inhabitants ρ = 0.421 (p = 0.091) (Figure 2c). On the other hand, the correlation of the numbers of CTs with other socio-economic parameters related to the economic condition of the region (unemployment rate). In this case a good negative association, with limited statistical significance was detected ρ = −0.8 (p = 0.104). Data are shown in Supplementary Material, Table 5.

Regarding the descriptive parameters of the level of NHS funding, we also collected data on health expenditure, NHS funding and pharmaceutical expenditure for the year 2022 and the corresponding data on the number of CTs on a regional basis (Supplementary Material, Table 4).

**TABLE 4:** Number of public and private IRCCS in Italy on Regional basis.

Region	Pubblic IRCCS	Private IRCCS	Total
LOMBARDY	5	13	18
LAZIO	2	7	9
EMILIA-ROMAGNA	5	1	6
VENETO	1	3	4
FRIULI-VENEZIA GIULIA	2	0	2
LIGURIA	2	0	2
TUSCANY	1	1	2
CAMPANIA	1	1	2
APULIA	2	1	3
SICILY	0	2	2
MARCHE	1	0	1
BASILICATA	1	0	1
PIEDMONT	0	1	1
ABRUZZO	0	0	0
CALABRIA	0	0	0
SARDINIA	0	0	0
UMBRIA	0	0	0

**FIGURE 3:**
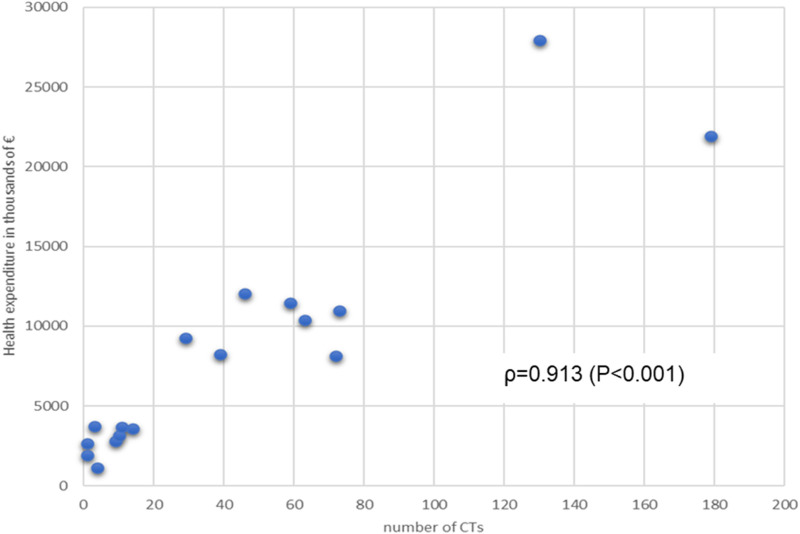
Relationship between Regional annual Health expenditure (financing of RHS) and number of CTs per Region. Spearman’s association value and p value are shown.

We determined the association data between the number of CTs and the macroeconomic parameters related to RHS, with the following results: RHS expenditure vs. number of CTs ρ = 0.913 (p < 0.001) (Figure 3), and number of CTs vs pharmaceutical expenditure (ρ = 0.036, p = 0.891) (graphs not shown).

**FIGURE 4:**
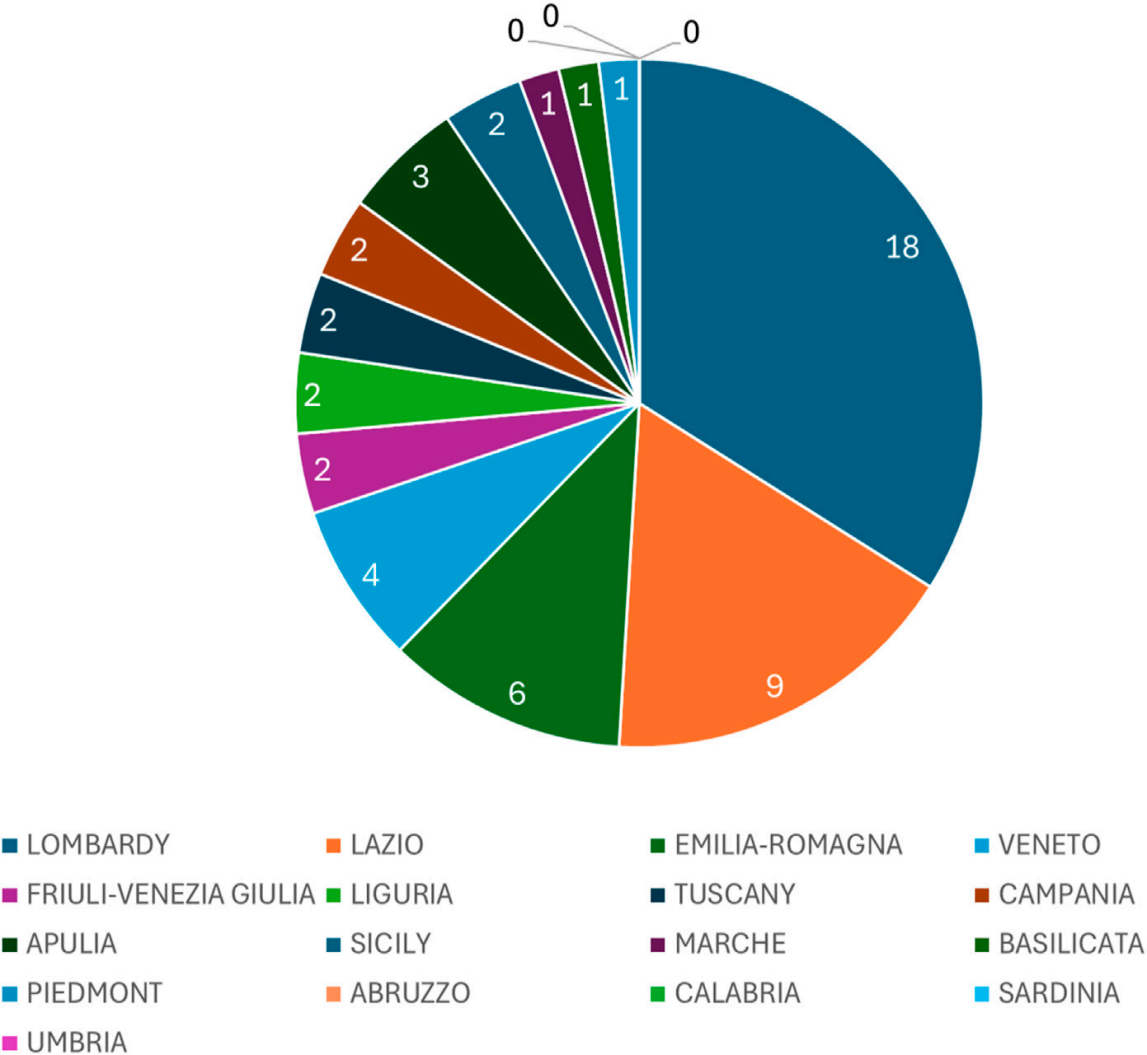
*Pie chart illustrating the distribution of IRCCS (public and private) across Italian regions.* Each slice represents the total number of IRCCS (Istituti di Ricovero e Cura a Carattere Scientifico) facilities present in each region, reflecting their territorial concentration.

We also investigated the key role of IRCCS in the increasing the number of gastroenterological clinical trials on regional basis. The number of IRCCS (public and private) are represented in Table 4, and plotted in a pie chart (Figure 4).

The analysis of the relationship between IRCCS (Institutes for Research, Hospitalization, and Healthcare) and the number of CTs (Territorial Centers) was performed using Spearman’s correlation coefficient. This non-parametric method is particularly suited for evaluating monotonic relationships without assuming a specific distribution of the data. The results reveal significant associations between the number of CTs and several variables related to IRCCS presence and characteristics across Italian regions.

For instance, the total number of IRCCS facilities (both public and private) shows a strong positive correlation with the number of CTs (ρ = 0.837, p < 0.001), showing the key role in shaping the regional healthcare landscape. Additionally, the binary variable “IRCCS Presence” (1 if at least one IRCCS exists in the region, 0 otherwise) correlates moderately with the number of CTs (ρ = 0.651, p = 0.005), highlighting the importance of having any IRCCS facility in influencing the availability of territorial health services.

Overall, the results indicate that IRCCS institutions contribute substantially to the organization and availability of healthcare services at the territorial level, reinforcing their importance as key drivers of regional healthcare systems.

Finally, a regression analysis was performed to evaluate the associations between number of clinical trials and regressors specified in the methods. Notably, inhabitants (x 100.000), population density and health expenditure returned significant positive associations (P < 0.05), by providing rate bigger than 1, equal to 1.037, 1.009 and 1.181, respectively (see Table 5). Oppositely, unemployment was inversely associated, by returning a significant rate equal to 0.899.

**TABLE 5:** Results by bias reduction-based regression analysis(i.e., Poisson regression models) on associations with the number of clinical trials per region Note. The response variable is the number of clinical trials, the statistical units are the n = 20 Italian regions. The significant results (P < 0.05) are shown in bold. Rate is defined as exponential transformation of the slope coefficient associated to regressor in the Poisson model. n: sample size; 95% CI: 95% confidence interval.

Regressor (n = 20)	Rate P-value 95%CI
Inhabitants (x 100,000, i.e., regional population)	1.037 <0.001 1.028; 1.045
Population density (number of regional inhabitants per km^2^)	1.009 <0.001 1.006; 1.011
Unemployment (%)	0.899 0.013 0.827; 0.978
Health expenditure (x 1000) (€)	1.181 <0.001 1.137; 1.226

## Discussion

This study, carried out on the 20 Italian regions, involved 103 CTs, conducted in 630 Clinical Centers across the country, was focused on the field of gastroenterological oncology, based on the evidence that there is a strong association between territorial socio-economic parameters and the ability to perform CTs. Moreover, the indicator of costs for the RHS also show a very good association with the number of active trials. As shown in Table 1, CTs are not evenly distributed across the country (Median = 12,5 and Interquartile Range (IQR) = 50,5), but sure more numerous in areas of central northern Italy than in the south and on the islands, as shown in our previous research works.

The distribution of clinical trials (CTs) across Italian regions reveals significant disparities influenced by socio-economic, demographic, and infrastructural factors. This analysis focuses on the relationship between the number of CTs, regional income levels, population density, healthcare expenditure, and unemployment rates. By examining these variables, we aim to understand the underlying dynamics that shape the allocation of clinical research resources in Italy.

Income levels play a crucial role in determining the availability of CTs. Regions with higher average incomes, such as Lombardy (€41,428) and Emilia-Romagna (€42,278), exhibit a significantly higher concentration of CTs, with 179 and 63 trials, respectively. Conversely, southern regions like Calabria (€26.603) and Basilicata (€30,420) report fewer trials, with only three and 4 CTs, respectively. Spearman’s correlation analysis confirms a moderate positive relationship between regional income and the number of CTs (ρ = 0.387, p = 0.124). This suggests that wealthier regions are better equipped to support clinical research infrastructure, attract funding, and engage in advanced medical innovation.

However, the relationship is not solely driven by income. For instance, Liguria, despite having a lower average income (€34,487), reports 14 CTs, indicating that other factors, such as population density and healthcare infrastructure, also influence trial distribution.

Population density emerges as another critical determinant of CT distribution. Regions with higher population densities, such as Lombardy (420 inhabitants/km^2^) and Lazio (332 inhabitants/km^2^), tend to have more CTs. This trend aligns with the logistical advantages of conducting trials in densely populated areas, where patient recruitment is more efficient. Spearman’s correlation coefficient for population density and CTs is strong (ρ = 0.777, p < 0.001), underscoring the importance of urbanization in shaping clinical research opportunities.

Conversely, sparsely populated regions like Valle d’Aosta (38 inhabitants/km^2^) and Molise (65 inhabitants/km^2^) report minimal CT activity, with only 0 and one trials, respectively. These findings highlight the challenges faced by rural areas in accessing cutting-edge healthcare services and participating in clinical research.

Healthcare expenditure further explains the uneven distribution of CTs. Regions with higher health spending, such as Lombardy (€21,907.56 million) and Lazio (€12,052.78 million), dominate the clinical trial landscape. The correlation between healthcare expenditure and CTs is very good associated (ρ = 0.7, p = 0.233), emphasizing the critical role of financial investment in fostering research capabilities.

In contrast, regions with lower healthcare budgets, such as Molise (€709.3 million) and Basilicata (€1,147.54 million), struggle to establish a comparable number of CTs. This disparity highlights the need for targeted investments to bridge the gap between economically advantaged and disadvantaged regions.

Unemployment rates present an inverse relationship with CT distribution. Southern regions, characterized by higher unemployment, such as Calabria (15.2%) and Sicily (14.3%), report fewer trials compared to northern regions like Lombardy (5.6%) and Veneto (5.2%). Spearman’s correlation analysis reveals a weak negative association (r = −0.2, p = 0.783), suggesting that economic instability may hinder clinical research activities (lowering the number of CTs). High unemployment often correlates with reduced healthcare access and limited institutional capacity, creating barriers to participation in clinical trials.

IRCCS institutions play a pivotal role in enhancing clinical trial opportunities. There are currently 51 IRCCS (Ministry of Health, 2020) in Italy (21 public and 30 private), with a clear prevalence in the centre-north (30 in the north and 11 in the centre) while only 10 are present in the south and islands (7 in the south and three in the islands). Regions hosting multiple IRCCS facilities, such as Lombardy and Emilia-Romagna, demonstrate a higher concentration of CTs. These institutions not only conduct research but also foster collaboration between public and private entities, driving innovation and improving healthcare outcomes. The presence of IRCCS is particularly evident in central and northern Italy, where their impact is most pronounced.

These findings suggest that the presence and type of IRCCS institutions significantly impact the distribution of CTs across Italy. Regions with a greater number of IRCCS, are more likely to have a higher presence of CTs (ρ = 0.837, p < 0.001). This could reflect a strategic alignment between advanced healthcare infrastructure and local healthcare needs, where regions prioritize establishing comprehensive healthcare networks by leveraging both public and private resources. Furthermore, the correlation between IRCCS presence and CT distribution underscores the critical role these specialized institutions play in enhancing regional healthcare accessibility and resource allocation.

Table 5 presents the results of a Poisson regression analysis (n = 20) evaluating the relationship between four regional predictors and an outcome variable, likely a count-based event. All predictors demonstrated statistically significant associations, though the direction and magnitude of effects varied. Inhabitants (per 100,000 population) showed a strong positive association with the outcome, yielding an incidence rate ratio (IRR) of 1.036 (calculated as 1036/1000, reflecting scaling per 100,000 population). This indicates that for every additional 100,000 inhabitants, the number of CTs increases by 3.6% (95% CI: 1.028–1.045, p < 0.001). Similarly, population density (per km^2^) exhibited a significant positive effect, with an IRR of 1.009 translating to a 0.9% increase in the outcome rate per additional thousand inhabitant per km^2^ (95% CI: 1.006–1.011, p < 0.001). Both population-related variables displayed narrow confidence intervals, underscoring precise estimates. Conversely, unemployment (%) was inversely associated with the outcome, with an IRR of 0.895 (95% CI: 0.821–0.975, p = 0.011). Health expenditure (per €1,000) emerged as the strongest positive driver, with an IRR of 1.180 (1180/1000), indicating an 18% increase in the number of CTs per additional 1,000 M€ spent (95% CI: 1.137–1.226, p < 0.001).

The findings highlight the critical role of regional demographics and healthcare investment in influencing the outcome. Population size and density likely reflect broader sociodemographic dynamics, such as resource demand or transmission opportunities (if the outcome is disease-related). Health expenditure’s robust association may signal the importance of infrastructure or access to services. Unemployment’s negative effect could imply economic stressors reducing the likelihood of the outcome or confounding factors (e.g., migration patterns). Notably, the IRRs for scaled predictors (population, health spending) require careful interpretation to avoid overestimating effects. While the model identifies significant predictors, limitations include a small sample size (n = 20), which may affect generalizability. Future studies could validate these associations with larger datasets and explore contextual factors, such as regional policies or environmental variables, to refine causal interpretations. These results hold policy relevance, particularly for prioritizing healthcare funding and addressing unemployment’s indirect impacts on public health or economic outcomes.

The study suggests that the number of active clinical trials (CTs) could serve as a novel benchmark for evaluating healthcare performance, as it strongly correlates with socio-economic factors and NHS efficiency. By integrating parameters like regional wealth, NHS expenditure, and population density, a unified index could provide a comprehensive measure of healthcare system effectiveness. This index would help policymakers identify disparities, allocate resources more equitably, and promote clinical research across all regions, ultimately improving healthcare outcomes and reducing inequalities.

We can hypothesise many additional reasons, difficult to measure, for this phenomenon, such as the different presence of knowledge opinion leaders (KOL), the greater and easier mobility of northern Italian areas compared to those in the south and the islands (Greco, 2019; Fattore et al., 2014; Sergi Bruno et al., 2020).

This study provides a comprehensive geographical analysis of clinical trials distribution in Italy, highlighting strong associations with economic and healthcare parameters. It emphasizes the role of IRCCS institutes and offers operable insights for policy interventions to reduce regional disparities and improve access to innovative therapies.

We have seen that, partly due to the regionalization of the NHS, the distribution of CTs across the country is extremely clustered. Also, the IRCCS play a key role in the conduction of CTs and, being unevenly distributed across the country, clearly contribute to the clustering of clinical research in Italy, in fact we have observed that, alone, the north carries out more than half of the CTs under examination. The clustering and the profound differences between areas in northern and southern Italy have many causes but two obvious ones certainly stand out, namely, the “wealth” of the RHSs in terms of public funding on healthcare and the wealth of the territories measured in terms of average wealth *per capita*. The failure to activate CTs (particularly promoted by Industry) in areas of the central south, with the proven negative effects on the sustainability of the SSN, in terms of missed opportunity for some areas of our country, sustains the phenomenon of health tourism (deleterious to regional coffers) and does not allow “democratic” access to innovative therapies that may be potentially effective in patients with GI malignancies. This analysis, in fact, collect and study, many different parameters that should be studied individually and could offer a perspective for conducting targeted actions by the central Government. Moreover, the CT data are accessible to all researchers and depend little on the method used in their determination, compared to macroeconomic data such as the average *per capita* wealth (strongly influenced by the type of data used in the calculation) or the unemployment rate. Even the NHS expenditure parameters do not consider the amount of out-of-pocket spending by citizens.

The utilisation of regional means may obscure local variations, and the study cross-sectional design imposes limitations on the ability to draw causal inferences. Furthermore, external factors such as patient mobility and private investments have not been thoroughly explored. In the present analysis, we explored regional disparities in clinical trial distribution, acknowledging the multifactorial nature of such differences. Among the variables that may influence the research landscape, educational status is often cited as a potential determinant of both public awareness and institutional readiness to engage in clinical studies. Populations with higher educational attainment may demonstrate greater health literacy, increased willingness to participate in clinical trials, and more favorable perceptions of biomedical research (Mills et al., 2006; Jenkins and Fallowfield, 2013). In parallel, regions with higher education indices often correlate with stronger academic institutions and infrastructures that can support trial conduction (Boulware et al., 2003).

Although we recognize the relevance of this variable, we chose not to incorporate educational status directly into our analysis. The decision was not based on its presumed irrelevance, but rather on methodological considerations. Data from the Italian National Institute of Statistics (ISTAT) provide educational breakdowns by multiple dimensions—age, sex, region, and qualification level—making harmonization and statistical interpretation complex. For instance, the dataset “Education levels of the resident population” divides the population into categories such as no education, lower secondary, upper secondary, and tertiary education, each with wide regional variability (ISTAT–Istituto Nazionale di Statistica, 2023b).

However, we acknowledge that education is a relevant social determinant and may mediate the relationship between regional development and clinical research capacity. Future investigations should consider this factor in combination with other socioeconomic indicators (e.g., deprivation index) to better understand how public knowledge, infrastructure, and institutional engagement shape research activity across territories. Such work may also support the development of policies aimed at reducing clinical research disparities by addressing structural and educational inequities. This study presents several inherent limitations that warrant careful consideration. First, the analysis was conducted at the regional level across the 20 administrative units of Italy, which imposes an intrinsic limitation due to the small sample size. Although the use of bias-reduced Poisson regression methods helped mitigate some of the statistical constraints related to small-n inference, we acknowledge that the number of observations limits the generalizability and robustness of the results. Nonetheless, since healthcare governance and resource allocation in Italy are highly decentralized, the region remains the most appropriate unit of analysis for policy-relevant evaluations of healthcare equity and trial distribution.

Second, the study relies on publicly available data from ClinicalTrials.gov, which, while comprehensive, may not capture all ongoing or recently completed studies, particularly those that are early-phase, observational, or institutionally sponsored. This potential registration bias could affect the completeness of the data. However, by limiting our inclusion criteria to interventional, pharmacological clinical trials in gastrointestinal (GI) oncology, we sought to reduce variability and ensure that the included studies adhere to standardized registration practices and regulatory oversight. Trials in GI oncology are predominantly multicentric and industry- or academically-sponsored, and as such, are subject to stringent reporting requirements.

GI malignancies represent a highly prevalent and medically prioritized group of conditions in Italy, with substantial clinical research activity across the territory. Focusing on this domain allowed us to capture a homogenous subset of trials with comparable design complexity, resource requirements, and organizational frameworks. Expanding the analysis to include heterogeneous indications (e.g., neurological, cardiovascular, or rare diseases) would have introduced significant confounding due to differences in epidemiology, trial logistics, and sponsor typology, potentially diluting the validity of observed associations between economic indicators and trial activity.

Other unmeasured contextual factors may have influenced trial distribution but were not included due to data unavailability. For instance, inter-regional patient mobility, which is known to affect healthcare access and research participation, was not integrated into our models, although it may amplify disparities in access to innovation (Greco, 2019; Fattore et al., 2014). Similarly, private-sector investment, including corporate R&D expenditure and private hospital activity, was not systematically available across regions and was excluded. These factors represent promising avenues for future research aimed at further disentangling the structural determinants of clinical trial infrastructure in Italy.

Despite these limitations, the study provides a novel and policy-relevant assessment of how macroeconomic and institutional parameters shape regional disparities in clinical trial activity. Future studies incorporating a broader range of pathologies, longitudinal designs, and more granular private-sector data may help confirm and expand upon our findings. Specific interventions aimed at promoting the spread of CTs more uniformly across the country are needed to support and promote the qualitative growth of healthcare.

The associations identified could be studied in the future on a larger dataset to identify artificial intelligence tools for predicting the ability of a territory to plan clinical research.

## Data Availability

The datasets presented in this study can be found in online repositories. The names of the repository/repositories and accession number(s) can be found in the article/Supplementary Material. Raw data are available in supplemental materials.

## Abbreviations

CHE, Current Healthcare Expenditure; CT, Clinical Trials; CU, Certificazione Unica (Unique Certification); D.lgs, Legislative Decree; DCT, Decentralized Clinical Trials; DM, Ministerial Decree; EU, European Union; GCP, Good Clinical Practice; GDP, Gross Domestic Product; GI, Gastrointestinal; HAQ, Healthcare Access and Quality; NHS, National Health Service; IRAP, Imposta Regionale sulle Attività Produttive (Regional Tax on Productive Activities); IRCCS, Institutes of Hospitalisation and Care of a Scientific Character; ISTAT, Italian Statistics Agency; KOL, Key Opinion Leader; L, Law; LEA, Livelli Essenziali di Assistenza (Essential Levels of Care); MEF, Ministry of Economy and Finance; NHS, National Health Service; OECD, Organisation for Economic Co-operation and Development; RHS, Regional Health Service; VAT, Value Added Tax; WHO, World Health Organization.

## References

[B1] Berdigaliyev N. Aljofan M. (2020). An overview of drug discovery and development. Future Med. Chem. 12 (10), 939–947. 10.4155/fmc-2019-0307 32270704

[B2] Boulware L. E. Cooper L. A. Ratner L. E. LaVeist T. A. Powe N. R. (2003). Race and trust in the health care system. Public Health Rep. 118 (4), 358–365. 10.1093/phr/118.4.358 12815085 PMC1497554

[B3] Bruzzi S. Ivaldi E. Santagata M. (2022). Measuring regional performance in the Italian NHS: are disparities decreasing? Soc. Indic. Res. 159 (3), 1057–1084. 10.1007/s11205-021-02775-8 34483439 PMC8404030

[B4] Campostrini S. Carrozzi G. Severoni S. Masocco M. Salmaso S. WHO Migration Health Programme, Office of the Regional Director, WHO Regional Office for Europe (2019). Migrant health in Italy: a better health status difficult to Maintain—Country of origin and assimilation effects studied from the Italian risk factor surveillance data. Popul. Health Metrics 17, 14. 10.1186/s12963-019-0194-8 31675961 PMC6824084

[B5] CEOWORLD Magazine (2023). Health care index 2023. CEOWORLD Magazine. Available online at: https://ceoworld.biz/2025/06/17/ranked-the-healthiest-and-unhealthiest-countries-in-the-world-2025/.

[B6] DATABASE ISTAT (2023). DATABASE ISTAT. Available online at: http://dati.istat.it/Index.aspx?DataSetCode=DCCV_TAXDISOCCU1.

[B7] Fattore G. Petrarca G. Torbica A. (2014). Traveling for care: inter-regional mobility for aortic valve substitution in Italy. Health Policy 117 (1), 90–97. 10.1016/j.healthpol.204.03.002 24726508

[B9] Gazzetta Ufficiale (2011). Legislative Decree no. 68/2011 published in the Gazzetta Ufficiale n.109 del 12 may 2011. Available online at: https://www.gazzettaufficiale.it/eli/gu/2011/05/12/109/sg/pdf.

[B10] Gazzetta Ufficiale (2000). Legislative decree no. 56/2000 published in the gazzetta ufficiale N. 62 on 15 march 2000. Available online at: https://www.gazzettaufficiale.it/eli/gu/2000/03/15/62/sg/pdf.

[B11] Gazzetta Ufficiale (2003). Law no.131/2003 published in the gazzetta ufficiale on 10 June 2003. Available online at: https://www.gazzettaufficiale.it/eli/gu/2003/06/10/132/sg/pdf.

[B12] GBD 2016 Healthcare Access and Quality Collaborators (2018). Measuring performance on the healthcare access and quality index for 195 countries and territories and selected subnational locations: a systematic analysis from the global burden of disease study 2016. Lancet 391 (10136), 2236–2271. 10.1016/S0140-6736(18)30994-2 29893224 PMC5986687

[B13] GBD 2019 Diseases and Injuries Collaborators (2020). Global burden of 369 diseases and injuries in 204 countries and territories, 1990-2019: a systematic analysis for the global burden of disease study 2019. Lancet 396 (10258), 1204–1222. 10.1016/S0140-6736(20)30925-9 33069326 PMC7567026

[B14] Greco C. (2019). Moving for cures: breast cancer and mobility in Italy. Med. Anthropol. 38 (4), 384–398. 10.1080/01459740.201.1592171 30971146

[B16] ISTAT – Istituto Nazionale di Statistica (2023b). Indicatori demografici territoriali. Available online at: https://demo.istat.it.

[B17] Jenkins V. Fallowfield L. (2013). Reasons for accepting or declining to participate in randomized clinical trials for cancer therapy. Br. J. Cancer 89 (10), 1783–1788. 10.1054/bjoc.2000.1142 10839291 PMC2363224

[B18] Kosmidis I. (2023). brglm2: Bias reduction in generalized linear models. R. package version 0.9.2. 10.32614/CRAN.package.brglm2

[B19] Kosmidis I. Firth D. (2021). Jeffreys-prior penalty, finiteness and shrinkage in binomial-response generalized linear models. Biometirka 108, 71–82. 10.1093/biomet/asaa052

[B20] Kosmidis I. Kenne Pagui E. C. Sartori N. (2020). Mean and median bias reduction in generalized linear models. Statistics Comput. 30, 43–59. 10.1007/s11222-019-09860-6

[B21] LaFleur J. Tyler L. S. Sharma R. R. (2004). Economic benefits of investigational drug services at an academic institution. Am. J. Health Syst. Pharm. 61 (1), 27–32. 10.1093/ajhp/61.1.27 14725117

[B22] Martin S. Siciliani L. Smith P. (2020). Socioeconomic inequalities in waiting times for primary care across ten OECD countries. Soc. Sci. Med. 263, 113230. 10.1016/j.socscimed.2020.113230 32823046

[B23] Martino M. L. Lemmo D. Bianchi M. González Leone M. F. Donizzetti A. R. Freda M. F. (2024). Public cancer screening services and participation: what meanings in users' narratives to promote engagement? Nurs. Health Sci. 26 (3), e13146. 10.1111/nhs.13146 39072951

[B24] Mills E. J. Seely D. Rachlis B. Griffith L. Wu P. Wilson K. (2006). Barriers to participation in clinical trials of cancer: a meta-analysis and systematic review of patient-reported factors. Lancet Oncol. 7 (2), 141–148. 10.1016/S1470-2045(06)70576-9 16455478

[B25] Ministry of Health (2020). Istituti di Ricovero e Cura a Carattere Scientifico – IRCCS. Ministry Health. Available online at: https://www.salute.gov.it/portale/ricercaSanitaria/dettaglioContenutiRicercaSanitaria.jsp?id=794&area=Ricerca%20sanitaria&menu=ssn&tab=2.

[B26] Nante N. Guarducci G. Messina G. Fabrizio M. Urbani A. (2021). Hospital patients’ migration among Italian regions. Eur. J. Public Health 31 (3). 10.1093/eurpub/ckab165.344

[B27] Nolte E. McKee M. (2004). Does healthcare save lives? Avoidable mortality revisited. Research report. Nuffield Trust. Available online at: https://www.nuffieldtrust.org.uk/research/does-healthcare-save-lives-avoidable-mortality-revisited .

[B28] OECD (2021a). Health at a glance 2021: OECD indicators. Paris: OECD Publishing.

[B29] OECD (2021b). Health expenditure and financing. Paris: OECD Health Statistics.

[B30] Park S.-M. Vonortas N. S. (2022). Translational research: from basic research to regional biomedical entrepreneurship. Small Bus. Econ. 60 (4), 1761–1783. 10.1007/s11187-022-00676-9 38625332 PMC9425788

[B31] Pedersen H. Skliarova T. Attkisson C. C. Lara-Cabrera M. L. Havnen A. (2023). Measuring patient satisfaction with four items: validity of the client satisfaction questionnaire 4 in an outpatient population. BMC Psychiatry 23 (1), 808. 10.1186/s12888-023-05310-w 37936112 PMC10630992

[B32] Polignano M. G. Pasculli G. Trisolini P. Di Lorenzo M. A. Dalfino G. Giannelli G. (2022). Economic impact of industry-sponsored clinical trials in inflammatory bowel diseases: results from the national institute of gastroenterology Saverio de Bellis. Front. Pharmacol. 13, 1027760. 10.3389/fphar.2022.1027760 36483744 PMC9723131

[B33] R Core Team (2024). A language and environment for statistical computing. Vienna, Austria: R Foundation for Statistical Computing. Available online at: https://www.R-project.org/(accessed on September 26, 2024).

[B34] Rapporto (2023). Il monitoraggio della spesa sanitaria, anno 2023 - rapporto. Available online at: https://www.rgs.mef.gov.it/_Documenti/VERSIONE-I/Attivit--i/Spesa-soci/Attivit-monitoraggio-RGS/2023/IMDSS-RS2023.pdf.

[B35] Regulation (EU) (2014). No 536/2014 of the european parliament and of the council of 16 April 2014 on clinical trials on medicinal products for human use, and repealing directive 2001/20/EC text with EEA relevance, art. 2. Par. Available online at: https://eur-lex.europa.eu/homepage.html?lang=en

[B36] Regulation (EU) (2019). “2019/1700 of the european parliament and of the council of 10 October 2019 establishing a common framework for european statistics relating to persons and households, based on data at individual level collected from samples, amending regulations (EC) no 808/2004, EC,” in No 452/2008 and (EC) no 1338/2008 of the European parliament and of the council, and repealing regulation (EC) no 1177/2003 of the European parliament and of the council and council regulation (EC) no 577/98.

[B37] Reinhardt U. Cheng T. (2000). The world health report 2000 – health systems: improving performance. Bull. World Health Organ 78 (8), 1064. Available online at: https://iris.who.int/handle/10665/268209

[B38] Rocha J. V. M. Santana R. Tello J. E. (2021). Hospitalization for ambulatory care sensitive conditions: what conditions make inter-country comparisons possible? Health Policy Open 2, 100030. 10.1016/j.hpopen.2021.100030 37383514 PMC10297774

[B39] Sanmarchi F. Esposito F. Bucci A. Toscano F. Golinelli D. (2022). Association between economic growth, mortality, and healthcare spending in 31 high-income countries. Forum Health Econ. Policy 24 (2), 101–118. 10.1515/fhep-2021-0035 36259392

[B40] Sergi Bruno S. D'Aleo V. Arbolino R. Carlucci F. Barilla D. Ioppolo G. (2020). Evaluation of the Italian transport infrastructures: a technical and economic efficiency analysis. Land use policy 99, 104961. 10.1016/j.landusepol.2020.104961 32834329 PMC7434470

[B41] Shen L. J. Chou H. Huang C. F. Chou G. M. Chan W. K. Wu F. L. L. (2011). Economic benefits of sponsored clinical trials on pharmaceutical expenditures at a medical center in Taiwan. Contemp. Clin. Trials 32 (4), 485–491. 10.1016/j.cct.2011.04.003 21530679

[B42] Signorelli C. Fara G. M. Odone A. Zangrandi A. (2017). The reform of the Italian constitution and its possible impact on public health and the national health service. Health Policy 121 (1), 90–91. 10.1016/j.healthpol.2016.10.008 27913055

[B43] Signorelli C. Odone A. Oradini-Alacreu A. Pelissero G. (2020). Universal health coverage in Italy: lights and shades of the Italian national health service which celebrated its 40th anniversary. Health Policy 124 (1), 69–74. 10.1016/j.healthpol.2019.11.002 31812325

[B44] Simonet D. (2011). The new public management theory and the reform of european health care systems: an international comparative perspective. Int. J. Public Adm. 34 (12), 815–826. 10.1080/01900692.2011.603401

[B45] Toth F. (2016). The Italian NHS, the public/private sector mix and the disparities in access to healthcare. Glob. Soc. Welf. 3, 171–178. 10.1007/s40609-016-0072-2

[B46] WHO (2016). Global health observatory data repository. Geneva: World Health Organization.

[B47] WHO (2024). WHO - the world health data hub. Available online at: https://data.who.int/countries/380#:∼:text=Life%20Expectancy%20Italy%20Male%20and%20Female.

[B48] Worsley C. Webb S. Vaux E. (2016). Training healthcare professionals in quality improvement. Future Hosp. J. 3 (3), 207–210. 10.7861/futurehosp.3-3-207 31098228 PMC6465816

